# Media Coverage of Pedophilia and Its Impact on Help-Seeking Persons with Pedophilia in Germany—A Focus Group Study

**DOI:** 10.3390/ijerph19159356

**Published:** 2022-07-30

**Authors:** Daniela Stelzmann, Sara Jahnke, Laura F. Kuhle

**Affiliations:** 1 Institute of Sexology and Sexual Medicine, Charité—Universitätsmedizin Berlin, 10117 Berlin, Germany; laura.kuhle@charite.de; 2 Institute for Computer Science, Freie Universität Berlin, 12165 Berlin, Germany; 3 Department of Health Promotion and Development, University of Bergen, 5003 Bergen, Norway; sara.jahnke@uib.no

**Keywords:** pedophilia, media coverage, media effects, stigma, prevention

## Abstract

The public stigma associated with pedophilia, the sexual attraction to prepubescent children, is tremendous. Previous research indicates that undifferentiated media coverage plays an essential role in perpetuating the public stigma by falsely equating pedophilia and child sexual abuse (CSA) and thus may stop persons suffering from a pedophilic disorder from seeking professional help. Until now, a comprehensive examination of positive as well as negative media effects on affected individuals is missing. Therefore, the present study explores if and how media coverage impacts the lives of help-seeking persons with pedophilia by conducting four qualitative focus group discussions with a clinical sample (N = 20) from the German Prevention Network “Kein Täter werden”. Present results demonstrate that media coverage of pedophilia was perceived as mostly undifferentiated, even though participants observed an increase in fact-based reporting over the years. Moreover, it seems that media coverage has strong emotional and behavioral consequences for patients (e.g., negative reporting reduced self-esteem). In sum, our results highlight that differentiated media coverage could play a key role in supporting help-seeking persons with pedophilic disorder, while the impact of undifferentiated media coverage appears to be mostly negative. Therefore, our results point to the need to reframe pedophilia using differentiated media coverage to help affected persons receive treatment efficiently and thereby prevent CSA.

## 1. Introduction

Studies indicate that approximately 1 to 5% of the male population have a sexual preference for prepubescent children (pedophilia, [1,2,3,4,5,6]). In contrast to common misperceptions, people with pedophilia do not necessarily commit child sexual abuse (CSA). This means that individuals with pedophilia can (and oftentimes do) live offense-free with full behavioral control [7]. Nevertheless, the sexual preference for prepubescent children represents an important risk factor for (repeated) sexual offending against children [8,9]. Moreover, there are individuals with pedophilia who perceive themselves at risk of committing CSA and/or experience distress caused by their sexual preference which motivates them to seek preventive treatment [1,10]. To support help-seeking individuals, various treatments have been installed nationally and internationally to offer help to affected persons (e.g., [11,12]). Yet, as these services rely on self-referral, there are concerns that potential clients refrain from seeking help because of the stigma associated with the sexual preference for children [13,14]. Thereby, previous studies indicate that media coverage of pedophilia plays an important role in perpetuating this stigma and instilling fear in people who are sexually attracted to children (e.g., [14,15,16,17,18,19]). However, these studies have largely focused on (non-clinical) community samples of individuals who tend to be well-adjusted and not at risk of committing CSA (e.g., [19]). Moreover, media effects on persons with pedophilia were examined marginally, focusing on most sensationalist and least fact-based examples of media reporting (e.g., [17]). As a consequence, potential positive and educational effects of media reporting on pedophilia have remained under-explored.

Relying on the model of reciprocal media effects, the present study aims to provide a comprehensive account of how people who are sexually attracted to children within a treatment program experience the media reporting on pedophilia. Based on data from four focus group interviews with patients with a pedophilic disorder (or in the case of hebephilia an unspecified paraphilic disorder) from the Berlin site of the German Prevention Network “Kein Täter werden”, the present study explored if and how media reports interfere with help-seeking behavior and the course of treatment in a population of persons with a sexual preference for children. Please note that in distinction to pedophilia, hebephilia describes the sexual interest by adults in pubescent children. As both groups are treated at the German Prevention Network “Kein Täter werden” because they are sexually attracted to children and as attraction patterns tend to overlap, the following research also included hebephilic clients. For more information on hebephilia, see [20,21].

### 1.1. Pedophilia

As previously mentioned, numerous studies indicate that about 1 to 5% of the male population is sexually attracted to prepubescent children [1,2,3,4,5,6], while the level of male sexual attraction to pubescent children (hebephilia) is more common (16.8% [4]; note that the sexual attraction to children appears to be considerably less frequent among women, [4,22]). Both sexual preferences for prepubescent (pedophilia) and pubescent (hebephilia) children are only considered pathological if the sexual attraction to children resulted in previous sexual offending against children, or causes significant distress and/or interpersonal difficulties (pedophilic disorder or unspecified paraphilic disorder [10]). Currently, there is little evidence showing that the sexual attraction to children can be “cured” in the sense that it can be converted into a sexual attraction to adults (teleiophilia; overview see [23]). Therefore, preventive treatment often focuses on behavioral control and/or the reduction of psychological distress. The German Prevention Network “Kein Täter werden” (translation: “Don’t offend”; also known as “Prevention Project Dunkelfeld”) was founded in 2005 to offer treatment to persons with a pedophilic disorder (or in the case of hebephilia persons an unspecified paraphilic disorder) [11,12]. Since 2018 it has been funded by the German Central Association of Health Insurance Funds (Spitzenverband Bund der Krankenkassen, GKV). Treatment goals within the prevention program involve increasing emotional self-regulation, control of sexual behavior, social functioning and empathy for CSA victims via cognitive behavioral interventions ([24], also see: [11,12,25]). Similar support services or projects have been established across Europe and America (e.g., “Prävention sexueller Kindesmissbrauch” (Germany), “Help wanted”, “The Global Prevention Project” (USA), “Stop-it-now” (USA, UK, and Netherlands)). However, treatment goals and needs can vary tremendously for individuals [26]. As Levenson et al. [27] point out, there are clients who neither need nor seek help with preventing sexual offenses against children but benefit from treatment to increase self-esteem and self-acceptance, reduce comorbid disorders such as depression, or dealing with loneliness or sexual frustration. Furthermore, pharmacological treatment, such as testosterone-lowering medication, can help clients who have problems to manage their sexual behavior toward children by reducing their sex drive [28,29].

### 1.2. Pedophilia and Stigma

According to Ervin Goffman, the term stigma describes “an attribute which is deeply discrediting” [30] (p. 3). In the case of pedophilia, previous research has documented higher levels of public stigmatization than for most other human characteristics, including alcoholism, schizophrenia and sexual sadism [31,32,33]. For instance, in a survey by Jahnke, Imhoff et al. [34], 14% of the German and 28% of the American respondents agreed that non-offending persons with pedophilia “should better be dead.”

Stigma constitutes a major source of stress for affected persons, even when the stigmatized status is not immediately apparent (e.g., for people with milder forms of mental disorders or people with non-heterosexual orientations [35,36]). Recent studies have documented that persons who are sexually attracted to children experience stigma-related stress [37,38,39,40], which is related to loneliness, psychopathological symptoms [38,39], and suicidal ideation [37]. Furthermore, persons with pedophilia also experience stigma in the health care sector, as many health care professionals report reluctance to offer treatment [41]. Hence, most persons with pedophilia choose to keep their sexual attraction a secret from everyone or nearly everyone [16,38,42]. Because of this, stigma may indirectly increase the risk of offending [43,44] via its assumed effect on the quality of life, mental health, fear of disclosure, and reduced therapy options for persons with pedophilia (for an overview see [13]).

### 1.3. Media Coverage of Pedophilia and Its Impact on Society

The low prevalence of pedophilia [1,2,3,4,5] and secret-keeping due to fear of rejection (e.g., [42]) make it unlikely that most people will knowingly have first-hand experiences with individuals with pedophilia. Nevertheless, as previously described, most people have a concept of individuals with pedophilia in their mind which is rather pejorative (e.g., [34]). Based on communication science theories such as the cultivation theory [45,46], it can be assumed that these individual concepts of pedophilia are derived from media coverage since it serves as second-hand experience by informing society about pedophilia. Therefore, persons with pedophilia as well as recipients depend on the media reporting about pedophilia accurately, thus shaping appropriate second-hand experiences [47].

Media coverage is determined by news factors and/or news values. This means that characteristics such as proximity, damage, or personalization structure the selection of events, and thus certain events are more likely to be reported than others [48,49]. Hence, media reporting on some topics may be biased ([48], also see [47]). To counteract this bias and to protect people who are the subject of media reporting, ethical journalistic standards are established in many countries worldwide [50,51]. For instance, in Germany, the so-called “Pressekodex” (translation: press code, [52]) includes guidelines to promote truthfulness and respect for human dignity as well as the protection of personal rights and due diligence with regard to journalistic investigation and communication of information. In addition, for particularly sensitive topics like CSA or suicide, separate recommendations (e.g., [53,54]) on how media reports should cover these topics to minimize negative effects (e.g., imitation effects such as the Werther effect [55]) exist. However, in the case of pedophilia and pedophilic disorder, comprehensive recommendations are currently lacking. Typical media coverage of pedophilia is characterized by primarily mentioning the subject in the context of severe and current cases of CSA (news factor: damage [48,49]). Thereby, media coverage tends to conflate pedophilia and CSA in the public consciousness [14,15,56,57,58]. For instance, Marc Dutroux, who was found guilty of sexually abusing and killing several children and teenagers in Belgium, was referred to as a “pedophile” in the media, even though his psychological evaluation found that he was not sexually attracted to children, but had other motives for his crimes (e.g., antisocial personality disorder, making profit by selling child sexual exploitation material; [59], also see [60]).

Although undifferentiated media coverage likely plays a role in shaping and perpetuating public stigma, media also has the potential to educate society about pedophilia [14,19,47]. According to health care professionals of the German Prevention Network “Kein Täter werden”, fact-based reporting on pedophilia exists (particularly in high-quality journalistic outlets) and contributes to spreading awareness about treatment options among the general public and potential clients. However, these reports primarily take place in exchange between medical experts from the German Prevention Network “Kein Täter werden” and journalists ([14], also see [61]). This underlines the importance of collaborations between medical experts in the field and media producers [62] by supporting journalists in the translation of complex and controversial topics “into content that can be understood by a layperson” [63,64,65] (p. 379). Ischebeck et al. carried out a study which found that journalists are willing to report differentiated article about pedophilia [47]. Their results demonstrate that the interviewed journalists were aware of the difference between pedophilia as a sexual preference and child sexual offending but the majority of the interviewed journalists assumed that most people who are sexually attracted to children are a danger to children. Even though the results show a positive attitude of journalists toward persons with pedophilia, the results may be biased by the left-liberal orientation of the publishers, for which a large part of the interviewed journalists worked ([47], also see [66]).

### 1.4. Media Coverage of Pedophilia and Its Impact on People with Pedophilia

Media effects that occur when protagonists are exposed to media coverage about themselves (or their social group) are referred to as reciprocal media effects [67,68]. The model of reciprocal media effects constitutes three fundamental assumptions: (1) In contrast to unaffected persons (bystanders, e.g., recipients), affected persons (protagonists, also media subjects) tend to show strong direct cognitive as well as emotional reactions and behavioral changes in response to the media coverage about themselves or their social group. (2) Moreover, protagonists are indirectly impacted by the reactions of recipients (e.g., peers or population in general) who receive the media coverage about the protagonists (or their social group) and thus adjust their attitudes and behaviors with respect to the protagonist (or their social group). (3) As a consequence, affected persons tend to speculate about media coverages’ impacts on recipients [67,68].

**Figure 1:**
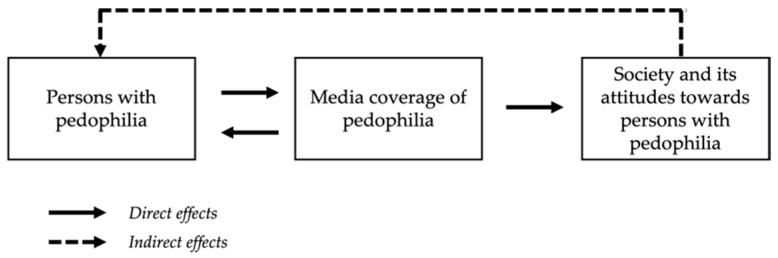
Reciprocal media effects on individuals with pedophilia as well as society (modified version of the model of reciprocal media effects [67,68]).

In the context of pedophilia, previous research supports these assumptions (illustrated in Figure 1). Prior study participants mostly described media reports on pedophilia and their effects on them in very negative terms [16,17,18,19]. In this context, participants mainly blamed media for the common misconception in society that falsely equates pedophilia and CSA (e.g., [15]). In isolated statements, this misconception is also seen as a danger that people with pedophilia are primarily exposed to a scenario “whereby offending is an inevitable conclusion” ([15], p. 10; also see [17]). Indeed, it is conceivable that most media coverage of pedophilia in the context of CSA causes help-seeking people with pedophilia to believe that they cannot be helped (e.g., [69]).

Furthermore, participants expressed that they felt intimidated when people with pedophilia are medially presented as “ticking time bomb[s]” [16] (p. 6) who will eventually commit heinous crimes against children (also see [15,18]). As a consequence, media coverage of pedophilia represents a daily stressor for some affected persons [18], which may contribute to fear and hopelessness [17]. Moreover, Ischebeck et al. [47] argue that undifferentiated media coverage may contribute to increased self-stigmatization, low mental health as well as increased risk to commit CSA since it may inhibit help-seeking behavior. Nevertheless, some participants described media coverage as a powerful tool to dispel existing barriers to seek help [19]. However, it should be noted that most prior studies that touched upon the effects of media reporting on persons who are sexually attracted to children did not focus on media effects in their research but were studying stigma. In this context, participants and researchers alike can be expected to pay more attention to negative aspects of media reporting. Furthermore, there is little evidence on affected individuals’ speculations about the impact of media coverage on recipients.

### 1.5. The Present Study

Help-seeking persons with pedophilia have unique experiences that are needed to gain a full understanding of the effects of media reporting, specifically relating to the uptake and course of treatment. Relying on the model of reciprocal media effects, our study was guided by the following overarching research questions:

- (RQ1) How do help-seeking persons with pedophilia perceive and evaluate the current media coverage about pedophilia?
- (RQ2) How does media coverage impact the everyday lives of persons with pedophilia, especially in the context of seeking and receiving therapeutic help?
- (RQ3) How should media cover pedophilia in a beneficial way from the affected point of view in order to reduce negative impacts?

To answer our research questions, we conducted four focus group interviews with 20 persons who received therapeutic help at the time of data collection at the Berlin site of the German Prevention Network “Kein Täter werden”.

## 2. Materials and Methods

### 2.1. Research Design

We decided to conduct qualitative focus group discussions with affected persons to enable them to express their views on the subject without limitations and thereby encourage each other to share their personal experiences [70]. Since the present research focuses on perceptions, evaluations, and experiences of help-seeking people with a pedophilic disorder (or in the case of hebephilia an unspecified paraphilic disorder) in treatment, the study used a purposive sampling method to gain comprehensive information [71]. Therefore, the current study took place at the Berlin site of the German Prevention Network “Kein Täter werden”, which provides preventive treatment for people who are sexually attracted to children (e.g., [11,12,25]). The present study was approved by the Institutional Review Board of the Charité—Universitätsmedizin Berlin, where participants were assessed and treated. In addition, ethical considerations were discussed with the respective therapists and institutional members. To ensure that the participants felt safe, the focus group discussions were guided by the therapists. Furthermore, the therapeutic setting of the research allowed participants to receive immediate assistance in the case that they were feeling distressed by the subjects discussed in the focus group sessions. Nobody—neither the therapists nor the participants—expressed reservations or withdrew from the study, and all participants gave written informed consent to participate in the study. The research design is followed by the Standards for Reporting Qualitative Research (SRQR, see [72]).

### 2.2. Partcipants

At time of the data collection, all participants were diagnosed with a pedophilic disorder (or in the case of hebephilia an unspecified paraphilic disorder) and joined a weekly group therapy session at the treatment site. To protect the identity of the participants, the authors assessed no demographic data such as age or profession. However, we know that patients at the Berlin site of the “Kein Täter werden” program are almost always male, with a median age of 37 years at the beginning of therapy and that up to 90% committed sexual offenses before entering therapy (mostly usage of child sexual exploitation material; [25]). All participants confirmed that they used the media regularly, mainly print journals, TV, the Internet, or specific social media to obtain entertaining and informing content.

### 2.3. Materials

We developed a semi-structured interview guideline including six topics: (1) description of general media usage unrelated to pedophilia, followed by (2) exposure to media coverage of pedophilia in their everyday life (e.g., How often are you exposed to media coverage on the subject?); (3) perception and evaluation of the media coverage of pedophilia (e.g., How would you describe the media coverage?); (4) impact of the media coverage of pedophilia on the motivation to seek treatment as well as (5) impact on the course of treatment (e.g., Are/were there moments when you talked specifically with your therapists about media coverage?); and ended with (6) a reflection of the ideal media coverage of pedophilia from the affected point of view (e.g., Is there any information that should be communicated in every article?).

### 2.4. Procedure

To ensure that the participants felt safe, the focus group discussions were moderated by the respective group therapists (*N* = 7). Accordingly, the therapists functioned as moderators in the respective focus groups. All therapists were specialized in the treatment of paraphilic disorders. The first author trained all seven therapists to guide the focus group discussion and familiarized them with the semi-structured interview guideline.

The focus group discussions about “Media coverage of pedophilia” with the participants took place as part of the regular group therapy session at the Berlin site of the German Prevention Network “Kein Täter werden”. To recruit participants, patients were asked in their regular group therapy session if they would be interested to participate in the study. All potential participants received detailed information about the study in a previous session. This process allowed the participants to ask for more information, express any concerns about the study or communicate beforehand if they rejected to participate with the respective therapists. Note that participants were informed that they could withdraw from the study at any moment and that refusal to participate would not lead to negative consequences for their treatment. However, nobody—neither the therapists nor the participants—expressed reservations or withdrew from the study. The discussions took place in April and May 2018 and lasted, on average, 1:26 h (01:12 h–01:40 h). The size of the four groups ranged between three and seven participants, representing a sufficient size and number of focus groups [73,74]. After each group discussion, the first author met with the therapists to reflect on the conversations and noted any specifics of the discussions. All group discussions were recorded by the respective therapists and transcribed afterwards. According to the Principle of Saturation, we closed the data collection after four focus group discussions [75] since we were able to identify new themes and sub-themes (inductive thematic saturation) as well as themes and sub-themes that were expressed in previous data (data saturation) [76]. 

### 2.5. Analysis

The analysis was conducted according to the qualitative content analysis technique of Mayring [77], using the software MAXQDAnalyse [78]. Firstly, the first author read the transcribed focus group discussions several times and selected potentially relevant text passages. Afterwards, the first author paraphrased and generalized all relevant statements and structured them into themes. Next, the first author embedded all identified themes in a broader context of meanings (e.g., quotes). The preliminary results were discussed with the third author in the first step. Subsequently, five of the therapists, who conducted the focus group discussions (at least one from every group), were invited to align the identified results and their perceptions of the group discussions. In both steps, the first author presented the identified themes and sub-themes including several anchor samples. If discrepancies occurred, the first and third authors revisited the corresponding themes and discussed their meaning until a high level of agreement was reached. Together with the first author, the second author reviewed the alignment between the themes and the results in the final manuscript, which led to further refinement of the themes. In sum, over 500 statements were coded. According to the research questions, three themes and ten sub-themes were identified (Table 1). Since the focus groups were originally conducted in German, the presented statements were translated by the first and second author.

## 3. Results

### 3.1. Perception and Evaluation of the Media Coverage (RQ1)

#### 3.1.1. Most Media Coverage on Pedophilia Is Undifferentiated, Some Media Coverage Is Fact-Based

Participants described the media reporting as undifferentiated across all four focus groups. From the participants’ points of view, this undifferentiated media coverage was mainly characterized by the focus on current CSA cases: “I also don’t remember [any news report] that has ever covered pedophilia without a concrete link to an abuse case” (participant 5, group 3). Several participants also noted that the media tends to focus their reporting on particularly severe CSA cases and “creates a kind of bogeyman” (participant 2, group 4). As such reports are highly emotionally charged, they leave little room for a nuanced discussion of pedophilia as a sexual preference, pedophilic disorder as a diagnosis, and sexual crimes: “In my opinion, the media coverage is lurid. If something horrible has happened, this doesn’t allow for distinctions (...). The whole spectrum of pedophilia [is] not covered at all. Instead, everyone is being lumped together” (participant 4, group 2).

According to participants, the equation of pedophilia and CSA obscured the fact that pedophilia is a sexual attraction “like homosexuality” (participant 4, group 2), which only constitutes one aspect of their identity or personality. From the participants’ points of view, the media neglects the important question of why people commit CSA and misses opportunities to spread awareness about where persons with pedophilia might find help: “Isn’t helping the [potential] offender [before they sexually offend] also helping victims?” (participant 4, group 3). 

Even though the participants mainly discussed unhelpful media coverage, members of each of the four focus groups mentioned examples of fact-based media reports that highlight the distinction between pedophilia and CSA. Focus group members noted that such reports often include information about prevention offers such as the German Prevention Network: “In [city name], [we] benefit from more frequent reporting about the project, and in such cases, the reporting is often very objective, and hardly any of the journalists (...) would dare to equate being pedophilic with being a child molester” (participant 1, group 1).

However, according to participants, this form of reporting is limited to high-quality media (especially public media) and on the sidelines (“(…) rather on late-night programs”, participant 1, group 3), meaning that only a small segment of the public can be reached.

#### 3.1.2. Media Coverage Has Become More Fact-Based over the Years

The participants noted a change toward more accurate reporting on pedophilia associated with the German Prevention Network: “A lot has happened in the last few years (...). They are broadcasting many good reports” (participant 5, group 1). Even if this development is too slow from most participants’ points of view, they were able to discern clear positive trends: “We had this case where a man abused and killed two little boys in a very headline-grabbing way, where, quote, unquote, ‘normal’ media actually mentioned that the man is quite obviously not pedophilic” (participant 1, group 1).

#### 3.1.3. Media and Public Agenda Block a Helpful Discourse on Pedophilia

Some focus group members were critical of the restraints put on the work of journalists, which they assumed negatively affects the quality of journalistic reporting: “Only bad news are good news” (participant 4, group 2). Participants believed that more spectacular and emotion-laden news generate more interest among readers, leading to more “clicks”. Hence, some acknowledged that it is difficult for journalists to do the topic justice and expressed sympathy for journalists who try to report on pedophilia accurately: “To say the least, I don’t think it’s that easy to take up the topic either” (participant 4, group 3). Others speculated that the public might have a need for a “maximum level of simplification” in order to “make it [pedophilia] more manageable” (participant 1, group 3). There was a broad consensus among the clients that only a few members of the general public would think that “a person with pedophilia [who] has done nothing wrong [is] a valuable member of this society” (participant 5, group 3). From their point of view, the “willingness of the masses” (participant 3, group 1) to deal with the topic in a differentiated way is lacking, which is why more accurate reporting would not make a difference for most people, even if there was more of it. They assumed that the media’s need to generate clicks and the audience’s need for simple messages that confirm their pre-existing views mutually reinforce one another, effectively blocking a differentiated public discourse on pedophilia. Therefore, individuals who want to swim against the current need to “grow a strong backbone, [and] be able to put up with a lot, including hostility” (participant 1, group 3) and are likely to be subjected to the question of whether they are “on the side of the child molesters or the side of the children” (participant 4, group 2).

### 3.2. Impact on Their Everyday Lives (RQ2)

#### 3.2.1. Undifferentiated Media Reports Trigger Negative Emotions and Threaten Self-Esteem

For many, undifferentiated and lurid media coverage triggered a negative reaction. Several participants reported that they tried to avoid media reports about pedophilia, mainly because they expected negative effects on their self-esteem and emotional state (typically heightened anxiety, rarely anger): “So due to headlines (…) when [pedophilia] was very much in the public eye, I also lost self-confidence and was more frightened” (participant 1, group 3). This had consequences for their behavior: For instance, some participants also mentioned that they did not actively seek out online content about pedophilia or comment on online articles because of privacy concerns, as their search and/or online profile may give away their sexual attraction to children to people: “I don’t notice it much, or more precisely, I don’t search for it. I’d be interested, but I have a bit of paranoia because, for instance, when I enter it into Google, it’s logged, saved, my IP address is known, so to speak” (participant 3, group 2). 

Moreover, participants remarked that social media user reactions such as “hate speech” or calls for “vigilante justice” (participant 1, group 3) scared them a lot. However, they also remembered some neutral or normal comments. Even though participants indicated that both, traditional as well as new media, gave them an impression of the current public opinions regarding their sexual attraction, only a few participants deliberately monitored the media coverage and user comments closely.

Participants stated that they regularly discussed current media reports in the therapy sessions because individual participants would share their distress about them with the group. These discussions helped them differentiate between media stereotypes and who they were as individuals: “Well, I don’t notice any influence either, definitely not a negative influence, because what is happening here [at the treatment center] is the exact opposite of what the media is reporting. We are approaching the topic calmly and objectively here (...)” (participant 2, group 3).

#### 3.2.2. Undifferentiated Media Reports Increase Barriers to Speak Openly about Pedophilia

While it was typical for clients to be secretive about their sexuality irrespective of current media reporting on pedophilia, many indicated that they were particularly careful during periods of intense reporting on CSA cases: “When such scandals come up or are blown out of proportion, then (...) I think, my God, for God’s sake, don’t out yourself in any way” (participant 4, group 1). Some revealed that they would delay their ’coming out’ until the media coverage subsided, in hopes that others would react more favorably at a later point: “If I have just noticed three reports in the newspaper during the week I plan to open up to another person, I might postpone it by a week or a month” (participant 3, group 2).

#### 3.2.3. For Some, Media Reporting on CSA Could Decrease the Risk of Criminal Acts

Our participants assumed that media coverage on CSA spreads awareness of CSA within society. Therefore, media reporting motivated some participants to change any (legal or illegal) behaviors that could out them as pedophilic: “You are a bit more aware that certain behaviors don’t work in public (...) because I immediately get strange looks when I walk around with a camera [to take pictures of clothed children and adolescents on the street]” (participant 2, group 2).

Media reports including information about the legal consequences, in particular, made them reconsider problematic behaviors. Some noted that this motivated them to avoid seeking and consuming child sexual exploitation materials: “(...) but the fear of discovery has reduced the consumption of child sexual exploitation material at least temporarily” (participant 4, group 2).

#### 3.2.4. Media Coverage Impacts Treatment Uptake in Opposite Ways

Participants identified advertisements for or fact-based reporting about the German Prevention Network “Kein Täter werden” as essential sources of information. Many stated that reports featuring individual patients from the program and more in-depth information about the goals and content of the program convinced many to seek treatment themselves: “(...) I also found it helpful to read interviews with treatment participants at this project or similar projects (…) who gave relatively positive accounts. I think that helped me build up trust to participate in the project myself” (participant 3, group 4).

On the other hand, a few participants stated that they were hesitant at first to seek help because they held stereotypical beliefs about other pedophilic individuals themselves. Expecting to meet and be associated with the pedophilic “monsters” (participant 2, group 2) from the media at the (group-based) treatment program was described as a sufficient threat to their self-image to delay treatment uptake: “I rather have the feeling that the more populist the reporting gets, the more difficult I find it to seek [treatment in a prevention program] (...). These headlines tend to trigger my insufficient acceptance of pedophilia (...) and then, for example, I also find it more difficult to come here” (participant 3, group 2).

### 3.3. Beneficial Media Coverage about Pedophilia (RQ3)

#### 3.3.1. Differentiation between Pedophilia and CSA

Participants suggested that it was most important for the media to stop equating pedophilia with CSA and instead “contribute to the public’s understanding that some have this sexual preference in an unbiased manner” (participant 5, group 2) as well as to “mention that one can get help” (participant 2, group 4). They also believed that fact-based media coverage should gain more prominence in mainstream media to reach and educate more people (e.g., during prime time). Participants were also hoping for more reporting on the many factors that increase individual risks to sexually offend besides being sexually attracted to children. They assumed this would make it easier for society in general and people who are sexually attracted to children, in particular, to differentiate between pedophilia and CSA: “It would be nice if society would eventually get to the point where we have normal reporting on the subject of pedophilia, both in terms of education and in terms of reaching, of course, potential offenders, the pedophiles themselves. Without equating these monstrous acts with all pedophiles” (participant 4, group 2).

#### 3.3.2. More Positive Examples

Participants also indicated that media outlets could portray more positive examples of persons with pedophilia who do not become offenders, even though they may have to “fight” (participant 2, group 1) every day to keep it that way. Furthermore, positive role models would help affected persons deal with their sexuality responsibly and lower barriers to seeking therapeutic help, if necessary: “Well, actually, I would just like to see a more positive image of us who are sitting here (…) who are (…) doing everything to avoid becoming abusive, that we are put more in focus than those who travel somewhere and then really become abusive (…). We just fall off the record, and that irks me a little” (participant 2, group 1).

Besides positive examples in general, participants would appreciate it if a prominent person ‘came out’ as pedophilic publicly and could serve as a role model, such as previous celebrities who came out as gay or shared their struggles with depression or substance addiction (e.g., Pete Doherty). Many mentioned the case of Sebastian Edathy, a member of the Bundestag (German Parliament), who allegedly possessed child sexual exploitation material. The participants were very critical of the damning media reporting on his case, which ended Mr. Edathy’s career and forced him to leave Germany. The prosecution was eventually halted without a conviction against the payment of a EUR 5000 fine [79]. Mr. Edathy admitted the possession of pictures of naked adolescent boys (of unclear legal status, as they may or may not have fulfilled the definition of child sexual exploitation material at the time) but denied sexual attraction to children [80]: “It is just (...) a pity that this happened, it was such an opportunity, where you have someone who is in the public eye, he can’t go anyway, he stands by his pedophilia, but then to be disappointed like this” (participant 2, group 1).

The participants also mentioned positive portrayals of persons who are sexually attracted to children. Yet, they criticized interviewees’ anonymous representations as problematic, even though they understand that their intent is to protect the protagonist: “On the one hand, I appreciate it when people (...) come out (...), but I always have a bit of a problem when [we are presented with] pedophiles with hoods, disguised voices, distorted [pixelated] images. (...) then I always have a bad feeling, then I think to myself (...) my God, they are monsters after all” (participant 4, group 1).

## 4. Discussion

The current study investigated how help-seeking persons with a pedophilic disorder (or in the case of hebephilia an unspecified paraphilic disorder) in a community-based CSA prevention program perceive and evaluate the media coverage on pedophilia (RQ1), how it impacts their everyday lives, especially in the context of seeking and receiving therapeutic help (RQ2), and how the media should ideally cover the topic (RQ3). In many regards, our results replicate previous findings on how participants who are sexually attracted to children from non-clinical community contexts perceive and react to media reports about pedophilia (e.g., [15,16,17,69]). However, our analyses also led to new insights regarding how media reporting both creates barriers for and facilitates the uptake of preventive treatment and may act as both risk and protective factors with regard to sexual offenses involving children or child sexual exploitation material. In the following, the results are discussed according to the research questions. 

### 4.1. Perception and Evaluation of the Media Coverage (RQ1)

In line with previous studies (e.g., [15,16,17,18]), our participants perceived the bulk of media coverage as undifferentiated and one-sidedly focused on CSA. In particular, participants lamented a lack of media coverage of the fact that a substantial number of people do not commit CSA despite being sexually attracted to them [7], and thereby neglects reporting positive portrayals of individuals with pedophilia [17]. 

Despite the fact that statements about undifferentiated media reports prevailed, there was consensus among the focus group members that fact-based or even sympathetic media portrayals of pedophilia exist and that they have become more common in recent years (examples of positive media coverage see [61]). The fact that positive media coverage was primarily mentioned in the context of reporting on the German Prevention Network “Kein Täter werden” underlines the importance of cooperation between medical experts in the field, public relations operators and journalists when working with a highly stigmatized patient group to render the subject intelligible [62]. Moreover, participants praised fact-based media coverage for raising awareness of treatment options, and some credited it as having convinced them to uptake treatment. Therefore, it seems plausible to assume that participants contacted the program because of the media coverage, not despite of it. A phenomenon which was already observed in previous studies on the link between mass media campaigns and health-related behaviors (e.g., [81]). This highlights that differentiated media coverage can motivate help-seeking persons who are sexually attracted to children (especially those who suffer from a pedophilic disorder or in the case of hebephilia from an unspecified paraphilic disorder) to self-refer to preventive treatment providers. This can be carried out by providing information about specific treatment offers. Not reporting on therapy options not only makes it more difficult for people with a sexual preference disorder to access help when they are in need but also suggests to the public as well as affected people themselves that they are untreatable and will inevitably sexually offend (e.g., [18,69]). However, improving the accuracy of media reporting on complex topics such as mental disorders often involves a continuous effort on both sides: the side of the treatment program and the media, which may last many years as results of recent media content analyses signal as well (e.g., [82,83]). Nevertheless, differentiated media coverage that refers to help offers seems all the more important to raise problem awareness and decrease therapy barriers for mental disorders in general as well as sexual preference disorders. 

Moreover, some of our participants presented very differentiated ideas about the interrelation of media coverage and public demand. It is difficult to say to what extent these ideas were influenced by the therapeutic process and might be an indicator of a cognitive strategy to regulate aversive emotions that might be elicited by stigmatizing media coverage. Additionally, these rationalizations might function as a coping strategy in order to establish a critical distance from media coverage to protect their self-esteem (e.g., Transactional Model of Stress and Coping [84]). Yet, besides their potential emotion regulation effect, their assumptions are also in line with prominent media theories such as the Agenda Setting Theory (see [85,86]). This theory proposes that both the media and audiences (public) have their “own” news agenda and that both agendas impact one another [86,87]. For this reason, it can be assumed that the quality of media reports does not solely depend on the framework conditions of the media but also on the public attitude toward the topic.

### 4.2. Impact on Their Everyday Lives (RQ2)

As reactions toward the perceived media coverage and in line with assumptions from the model of reciprocal media effects [67,68], participants across all focus groups indicated various changes on the emotional (e.g., anxiety caused by undifferentiated reports) as well as behavioral levels (e.g., abstinence from the use of child sexual exploitation material, media observation) in their daily lives. In fact, focus group members experienced links between undifferentiated media reports and increased expectations of rejection, internalized stigma, and concealment of identity. Many noticed a negative effect on their emotional state and self-esteem during phases of intense (and typically undifferentiated) reporting on pedophilia and CSA. In line with assumptions from stigma stress theories [88,89,90], it appears that undifferentiated and stigmatizing media coverage leads to distress (also see [91]). Remarkably, our data demonstrate that stigmatizing media coverage creates barriers to set up and run initiatives to offer preventive treatment to people who may be at risk of committing CSA and/or experience distress caused by their sexual preference. Even though participants were able to recognize that they were not ‘monsters’ as detailed in the media, media coverage still contributed to stereotypes about other people with this sexual attraction (also see [92,93]). This creates obvious barriers for a group-based program such as the “Kein Täter werden” network. As a result, some stated that they were hesitant about contacting the German Prevention Network because of their own (media-based) ideas about others who are sexually attracted to children. Once those affected have overcome this hurdle, treatment helped them to critically examine media reports and distinguish between the media framing and their personal identity. Within group therapy sessions, participants learned to correct their own assumptions about others who are sexually attracted to children as well as about themselves, which helped them become more resilient regarding the negative effects of undifferentiated media reporting on pedophilia on their self-esteem. Hence, it can be concluded that media literacy training poses an essential component within therapeutic settings to reduce adverse reciprocal media effects. Previous research on the association between media literacy and health promotion is in line with this conclusion (e.g., [94,95,96]).

Stinkingly, isolated statements demonstrate that media coverage contributed to law-abiding behavior by opening their eyes to the potential negative consequences of CSA and/or child sexual exploitation material for the involved children and also for themselves. To avoid these anticipated consequences, some participants adapted their behavior in order to be more in accordance with social norms, at least in the short term (furthermore, see [97]). Therefore, it was indicated that information about the legal consequences of child sexual offending can deter some people with a pedophilic disorder (or in the case of hebephilia an unspecified paraphilic disorder) from accessing child sexual exploitation material. Moreover, this not only affected the participants’ motivation to stop the use of child sexual exploitation materials but also the use of images of children that are not criminally relevant and are irrelevant for criminal prosecution. The fact that participants also refrained from legal options to achieve sexual gratification indicates that persons with pedophilia speculate about the impact of media coverage on recipients as postulated by the model of reciprocal media effects [67,68]. Nevertheless, future studies need more rigorous tests to establish whether there is a link between the awareness of legal consequences, the likelihood of decreasing problematic behaviors and speculated public reactions. 

Besides the impact of traditional media, future research should investigate the dynamics caused by new media. Our participants stated that because of privacy concerns and the risk of being outed based on their online behavior and profiles, they avoid being exposed to content about pedophilia online. Unfortunately, this avoidance strategy may also hinder people who are sexually attracted to children from gaining information about therapeutic options or even to look for help online with online treatment or self- management options developed for those who are too afraid to present themselves in person to treatment providers or who have no treatment providers in their local proximity. Moreover, present participants described being intimidated by social media phenomena such as hate speech characterized by incitement to hatred or calls for vigilante justice. A current study shows that social media has “not only become a fertile soil for the spread of hateful ideas but also motivates real-life action” [98] (p. 2164). A recent example is the social media attacks on the assistant professor and researcher Allyn Walker for pointing out the importance of destigmatizing pedophilia and differentiating between pedophilia and CSA for efforts to prevent CSA [99]. Accordingly, the online outrage on Twitter quickly escalated to serious threats against the life of this researcher. To counteract such phenomena, it seems necessary to encourage, for instance, moderate discussions or turn off the comment function on specific topics. However, social media use can also have positive effects (e.g., exchange with like-minded others [16]), which are little understood now.

### 4.3. Beneficial Media Coverage about Pedophilia (RQ3)

Present research demonstrates how valuable differentiated media coverage about pedophilia can be for people with this attraction. However, media coverage portraying role models who are sexually attracted to children but oppose CSA and have good behavioral control is lacking. In line with the exemplification theory, such personal portrayals could increase empathy toward people with pedophilia ([100]; also see [101])—an idea that was also endorsed by the present study participants. Moreover, this reporting style could help people with pedophilia find role models and develop a positive non-offending identity. Moreover, this kind of favorable media content can positively influence public attitudes towards stigmatized groups, which previous studies have shown [102,103,104].

Besides the impact on affected persons, we have to take into consideration that media coverage about pedophilia serves as second-hand experience for most people from the general population, where child-attracted people have few opportunities to talk openly about their sexual preference without receiving negative consequences [47]. Therefore, reframing pedophilia by using fact-based and differentiated media reporting is necessary to contribute to a more differentiated public discourse and counteract biased heuristics (e.g., [105]). As a first step, Stelzmann et al. [14] suggested that pedophilia should not primarily be reported on in a context-bound manner (e.g., current case of CSA; episodic framing [106]). Furthermore, these authors suggested that media should place a higher news value on community or clinical samples of persons with pedophilia as a group who is underrepresented in today’s media. Such media coverage would contribute to more fact-based and accurate portrayals of pedophilia (e.g., interviews with experts; thematic framing [106]). 

By focusing public attention on a small set of individuals who are regularly portrayed as extremely disturbed and sub-human, media coverage perpetuates common misperceptions or stereotypes [105,107,108,109]. Therefore, reframing pedophilia could help cover CSA in a more realistic light and may also increase public awareness about other types of child sexual offenders who do not have a sexual preference for children (e.g., [110]). In order to support journalists who report on pedophilia in a more differentiated way, to help affected persons in need of treatment to find treatment providers as well as to educate the public about pedophilia, it seems necessary to develop specific reporting guidelines. These can be based on existing guidelines, such as those on CSA or suicide, as well as input from researchers and clinicians. After all, studies indicate that there are journalists who are willing to report openly and in a differentiated manner on the topic [47,62,66].

## 5. Limitations

The present study documents the perspectives of persons diagnosed with a pedophilic disorder (or in the case of hebephilia an unspecified paraphilic disorder) who are involved in a preventive treatment program. Thus, our study cannot inform on the views of people who are pedophilic (or hebephilic) but do not fulfill the criteria for a pedophilic disorder (or an unspecified paraphilic disorder) or those who meet the criteria but do not seek treatment in the program (e.g., because they are too afraid to contact the German Prevention Network “Kein Täter werden” or do not live in close proximity of the treatment site). Furthermore, it is difficult to generalize the results from our German setting to other countries with different laws regarding the treatment of affected persons (e.g., [111]), different mental health systems and services (e.g., [112,113]), a different media system (e.g., [114]), and potentially an even more severe public condemnation of people who are sexually attracted to children (e.g., [34]). Additionally, our study does not reflect the views of persons with pedophilia who commit CSA or are at risk of doing so but are not problem-aware and therefore are not motivated to seek and receive treatment (theories of sexual offending, overview see [115]).

Furthermore, the study reflects the participants’ subjective perceptions, which may be colored by various factors (e.g., different media exposure (e.g., [116]), new perspectives and strategies gained in treatment (e.g., [117])). In addition, the group discussions took place within the therapy session, and it cannot be excluded that this setting leads to demand effects, where clients believe that they have to endorse certain views in order to protect the therapeutic alliance [118].

Moreover, in our study, a qualitative design was used to generate insights regarding a neglected research topic. By analyzing the data, statements of each individual were put into focus, while small group dynamics were neglected [119]. However, we identified nearly all themes across the four groups, which indicates that the results were not dependent on specific group dynamics.

## 6. Conclusions

Differentiated media coverage can play a key role in destigmatizing pedophilia in general (and differentiating it from a pedophilic disorder or an unspecified paraphilic disorder as well) while taking preventive action against child sexual exploitation. In contrast to previous research, our results indicate several positive effects of media reporting, which appeared to be mostly driven by differentiated media coverage. Nonetheless, our results also add to concerns voiced by previous authors (e.g., [14,18]) that media coverage of pedophilia is often counterproductive—even if there is evidence of improvement. Since “science often communicates in a more complex way than journalism (…) it’s important to explain scientific research to the public in an understandable way. That’s why good science communication is so important” [62] (pp. 213–214). Therefore, it seems essential for medical experts to proactively support journalists in addressing pedophilia and to ensure fact-based media coverage [42,120]. Previous scholars have suggested the use of low threshold offers such as fact boxes, guidelines [14,120] or (digital) contacted-based interventions [121,122,123] as a means to reach out to journalists as well as society.

## Figures and Tables

**Table 1:** Identified themes and sub-themes.

Themes	Sub-Themes	Examples
Perception and Evaluation of the Media Coverage	Most Media Coverage on Pedophilia Is Undifferentiated	“(...) the entire range [of pedophilia] is not represented [in the media]. It is about the worst [CSA] cases (...), the tip of the iceberg.”
	Some Media Coverage on Pedophilia Is Fact-Based	“30% [media reports] that are now informative, empathetic, (…) deal with the whole topic.”
	Media Coverage Has Become More Fact-Based Over the Years	“Yes, yes, I have a hunch that it has improved.”
	Media and Public Agenda Block a Helpful Discourse on Pedophilia	“The current form [of media coverage about pedophilia] has grown this way because society is the way it is.”
Impact on Their Everyday Lives	Undifferentiated Media Reports Trigger Negative Emotions and Threaten Self-Esteem	“Now that I think about it, maybe fear of being, um, punished or judged, rejected.”
	Undifferentiated Media Reports Increase Barriers to Speak Openly About Pedophilia	“(…) and the media have made a big contribution to the stigmatization, and that makes it even more difficult for pedophiles to come out.”
	For Some, Media Reporting on CSA Could Decrease the Risk of Criminal Acts	“Reports in the media definitely make you more cautious in how you deal with things (…)”
	Media Coverage Impacts Treatment Uptake in Opposite Ways	“Reports about this house, for example, brought me here somehow. Otherwise, I probably wouldn’t have been here [at the prevention network].”
Beneficial Media Coverage About Pedophilia	Differentiation Between Pedophilia and CSA	“[That] these two groups [sexual offenders vs. persons with pedophilia] in the end, that must arrive [in society], that they are not [necessarily] the same.”
	More Positive Examples	“So, I would also welcome it if (...) the reporting would take place on the basis of positive examples.”

## Data Availability

The data are not publicly available due to ethical restrictions.
